# Successful Treatment of Leukemic Synovitis Complicated With Chronic Myelomonocytic Leukemia

**DOI:** 10.1155/crh/3159757

**Published:** 2025-09-02

**Authors:** Hiroki Tsutsumi, Masahisa Kudo, Nobuo Maseki, Machiko Kawamura, Kazuhiko Kobayashi, Yasunobu Sekiguchi, Yuka Harada, Daichi Sadato, Hirofumi Kobayashi

**Affiliations:** ^1^Department of Hematology, Saitama Cancer Center, Saitama, Japan; ^2^Department of Clinical Laboratory Medicine, Saitama Cancer Center, Saitama, Japan; ^3^Department of Clinical Laboratory, Tokyo Metropolitan Cancer and Infectious Diseases Center Komagome Hospital, Tokyo, Japan

## Abstract

Chronic myelomonocytic leukemia (CMML) is a myeloproliferative disease characterized by monocyte-predominant hematopoiesis. It is sometimes complicated with cutaneous involvement known as leukemic cutis, which is associated with a poor prognosis. We report a patient with CMML who developed fever with knee joint swelling and pain. The patient was considered to have leukemic synovitis, and treatment with azacitidine improved her symptoms. Our case suggested that leukemic synovitis might indicate the indication for treatment, and arthrocentesis should be performed in patients with leukemia who present with joint swelling.

## 1. Introduction

Myeloproliferative neoplasm (MPN) is a disease caused by tumorigenesis at the level of hematopoietic stem cells and characterized by a marked proliferation of myeloid cells [1]. The WHO classification includes the category myelodysplastic syndrome/MPNs (MDS/MPNs) for myeloid tumors that have features of both MDS and MPN, including chronic myelomonocytic leukemia (CMML) [2].

CMML is known to cause systemic inflammatory autoimmune diseases, leukemia cutis, lysozyme-induced nephropathy, and pleural effusion in addition to common symptoms such as fatigue or malaise [3, 4]. Among these, leukemia cutis is seen in about 10% of cases [5, 6], and its complications have been reported to indicate a poor prognosis [7].

We report a case of synovitis of the knee that was thought to have originated from CMML. Infection, autoimmune disease, or crystal deposit arthritis could be ruled out, and treatment of CMML relieved the symptoms of joint swelling, suggesting a connection with the underlying disease. A diagnosis of leukemic synovitis was made.

## 2. Case Presentation

The patient is a 73-year-old woman who was referred by her primary care physician one month prior to admission with an elevated white blood cell (WBC) level. Her complete blood count (CBC) was WBC 16,110/μL (normal range: 3300–8600/μL) (myelocytes: 1.0%, metamyelocytes: 1.0%, neutrophils: 30%, eosinophils: 37%, basophils: 4.0%, monocytes: 14%, and lymphocytes: 13%), hemoglobin 8.6 g/dL (normal range: 11.6–14.8 g/dL), mean corpuscular volume (MCV) 96 fL (normal range: 83.6–98.2 fL), and platelets 196,000/μL (normal range: 158,000–348,000/μL). Her serum laboratory results showed lactate dehydrogenase (LDH) at 543 U/L (normal range: 124–222 U/L) and C-reactive protein (CRP) at 1.74 mg/dL (normal range: 0.00–0.14 mg/dL). All the other biochemical tests were within normal range.

**Figure 1:**
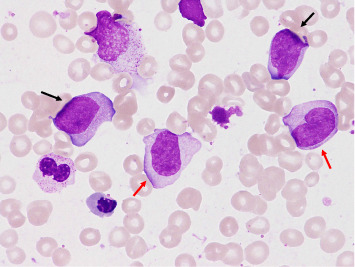
Initial presentation of the bone marrow examination. May–Grünwald Giemsa staining × 600. Blasts (black arrow) and monocytes (red arrow) appeared.

The bone marrow examination showed hypercellular marrow with 9.4% blasts, elevated monocytes (15%), megaloblastic change in some erythroblasts, multinucleated megakaryocytes, and Pelger–Huët anomaly and hypogranularity in some myeloid cells (Figure 1). *BCR*::*ABL1* or *PDGFRA* fusion genes were not detected. Next-generation sequencing revealed *SF3B1* K666N (variant allele fraction [VAF] 54.67%) and *SRSF2* P95R (VAF 50.18%) mutations.

As eosinophil counts in peripheral blood and bone marrow were elevated, atypical CML could be ruled out, and MDS/MPN with *SF3B1* mutation and thrombocytosis could also be excluded because of the absence of thrombocytosis. Based on the diagnostic criteria [2], the diagnosis of CMML was confirmed.

Since the patient was of advanced age, not suitable for hematopoietic stem cell transplantation, and not transfusion-dependent, she was initially treated as an outpatient on a wait-and-see schedule.

Two days prior to admission, the patient developed a painful swelling of the right knee without any history of trauma. She also developed fever and visited her family orthopedist but was advised to follow up. Because of the worsening of symptoms, she was admitted to our hospital as an emergency for close examination and treatment.

**Figure 2:**
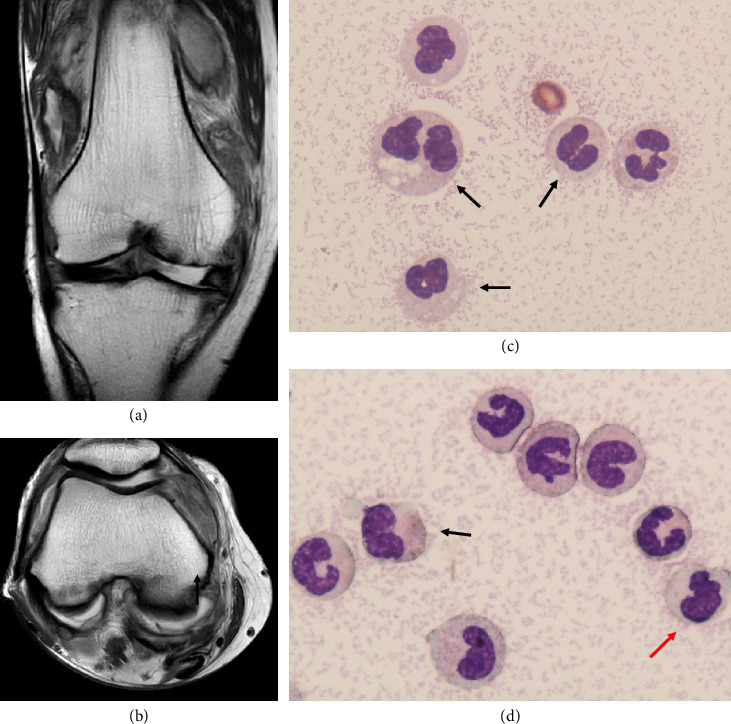
Coronal and axial magnetic resonance imaging of the right knee (T2WI high) of initial presentation (a, b). Synovial fluid examination. May–Grünwald Giemsa staining × 1000. Pseudo–Pelger–Huët cells (black arrow) and hypogranular neutrophils (red arrow) appeared (c, d).

On admission, her vital sign was body temperature 38.7°C, blood pressure 114/76 mmHg, and heart rate 108 bpm. Her physical examination was noted at right leg edema, with prominent right knee pain and swelling. Peripheral blood counts on the day of admission were as follows: WBC 31,600/μL (myelocytes: 2.5%, metamyelocytes: 1.5%, neutrophils: 80%, eosinophils: 0%, monocytes: 10%, and lymphocytes: 6%), hemoglobin 6.8 g/dL, MCV 97.3 fL, and platelets 62,000/μL. The biochemical tests were LDH 647 U/L, CRP 22.46 mg/dL, aspartate aminotransferase (AST) 27 U/L (normal range: 13–30 U/L), alanine aminotransferase (ALT) 15 U/L (normal range: 10–42 U/L), total bilirubin (T-Bil) 0.7 mg/dL (normal range: 0.4–1.5 mg/dL), blood urea nitrogen (BUN) 25 mg/dL (normal range: 8–20 mg/dL), creatinine (Cre) 1.35 mg/dL (normal range: 0.65–1.07 mg/dL), total protein (TP) 5.3 g/dL (normal range: 6.6–8.1 g/dL), and albumin (Alb) 2.5 g/dL (normal range: 4.1–5.1 g/dL). Coagulation tests were within normal range. Contrast-enhanced magnetic resonance imaging showed inflammatory changes primarily in the synovial membrane of the knee joint (Figures 2(a) and 2(b)). Deep vein thrombosis was ruled out because no obvious venous thrombus or blood flow disruption was found on contrast-enhanced computed tomography and lower limb venous ultrasound.

Based on these findings, we initially suspected pyogenic knee arthritis and performed a knee arthrocentesis, but a pale yellow, viscous fluid was withdrawn, and a similar procedure was performed four times, but bacterial cultures were repeatedly negative. No pyrophosphate or uric acid crystals were detected in the synovial fluid. Blood cultures were also negative. Autoimmune arthritis was also suspected, and various autoantibodies such as antinuclear antibodies were measured, but they were negative.

After hospitalization, we administered antibiotics and analgesics to her, but her symptoms further exacerbated. As the results of bacterial culture were repeatedly negative, the patient was treated with prednisolone (PSL) 40 mg (= 1 mg/kg) for symptomatic relief, and the swelling and pain in the knee joint improved.

On the 14th day of PSL, the dose was reduced to 20 mg, and the knee joint symptoms began to deteriorate. During the course of the disease, we suspected an exacerbation of the CMML from the elevation of WBC and LDH.

We performed knee arthrocentesis again, and a smear specimen of the puncture fluid showed granulocytes with Pelger–Huët anomaly and hypogranularity (Figures 2(c) and 2(d)). We hypothesized an association between the CMML and knee synovitis from these findings.

**Figure 3:**
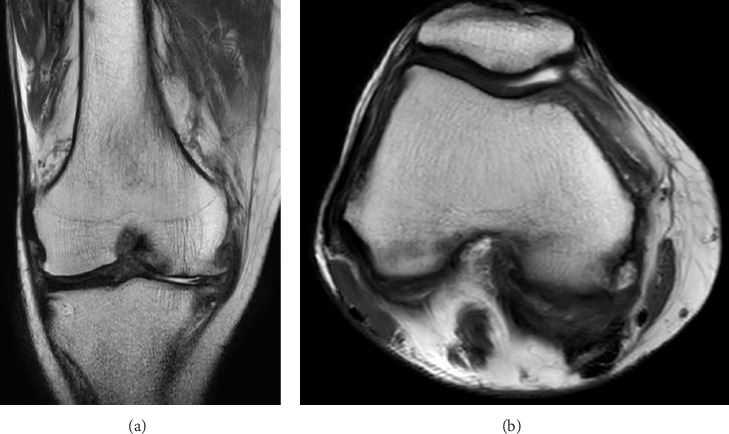
Coronal and axial magnetic resonance imaging of the right knee (T2WI high) after azacitidine treatment. Thickened synovium diminished after treatment (a, b).

**Figure 4:**
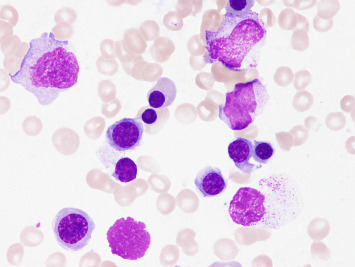
Reanalysis of the bone marrow examination after azacitidine treatment. May–Grünwald Giemsa staining × 600. Restoration of erythroblast and disappearance of blasts.

Because her peripheral monocyte count was over 1 × 10^9^/L and she had more than one lineage dysplasia in the bone marrow, we determined that she was eligible for azacitidine. After the start of treatment (75 mg/m^2^/d, 7 days), WBC and LDH decreased quickly, and the swelling of the knee joint gradually improved. MRI was reevaluated, and thickened synovium, the findings of synovitis, disappeared (Figures 3(a) and 3(b)). We performed bone marrow examination and confirmed the appearance of erythroblasts and the disappearance of blasts, indicating restoration of normal hematopoiesis (Figure 4). Based on these results, the diagnosis of CMML-associated leukemic synovitis was made.

After her blood counts recovered, she was discharged from the hospital and has been followed up on an outpatient basis with no worsening of the swelling in the right knee joint.

## 3. Discussion

We present a case of leukemic synovitis in a patient with CMML, which is known to cause leukemic cutis as an extramedullary manifestation, but leukemic synovitis was also manifested, and the patient's joint swelling was the initial indication for treatment.

Because of the diverse biological background of CMML, the treatment strategy for CMML is a watch-and-wait attitude with active monitoring, followed by therapeutic intervention with hypomethylating agents when symptoms such as blood transfusion dependence and/or constitutional symptoms appear. Allogeneic hematopoietic stem cell transplantation may be considered depending on age and comorbidities [8, 9].

In general, leukemia presents with a variety of extramedullary manifestations, especially in CMML, where leukemic cutis is reported to occur in about 10% of patients due to inherent plasticity between monocytes and macrophages [5, 6].

Other extramedullary manifestations, such as leukemia-related joint symptoms (leukemic arthritis [LA]), are reported to occur in approximately 4%–13% of leukemia patients [10]. This arthritis preferentially involves large joints and occurs at any time during the course of leukemia and may be the initial presenting symptom.

LA is defined as joint pain and swelling associated with leukemia after other causes of arthritis have been excluded [11].

The pathophysiology of LA is assumed to include direct infiltration of leukemic cells, bleeding in the joints, autoimmunity, and crystal-induced synovitis [12, 13].

Among LA, direct leukemic cell infiltration into the synovial membrane of joints is known as leukemic synovitis [14]. There have been a few case reports of leukemic synovitis [10, 15–26], and it is considered a rare complication.

In the present case, arthrocentesis revealed atypical granulocytes, including pseudo–Pelger's nucleus abnormalities, and the joint swelling improved with treatment of the CMML, which is consistent with the clinical presentation of leukemic synovitis.

In CMML, the prognosis of patients with leukemic cutis has been reported to be poor, suggesting disease progression and transformation to acute leukemia [7]. Leukemic synovitis is also part of extramedullary lesions and, like leukemic cutis, reflects the aggressiveness of the disease, indications for treatment, and the poor prognosis.

The patient has *SF3B1* K666N and *SRSF2* P95R mutations as genes involved in splicing. Although splicing mutations are usually considered mutually exclusive [27], exceptions have been reported [28–30]. Large-scale sequencing studies of hematologic malignancies have revealed that 0.85% harbored 2 concomitant bona fide splicing factor mutations across 4231 patients [31]. Clinical and morphological significances associated with double splicing factor mutations are to be determined [32].

We report a patient with CMML complicated with leukemic synovitis, and since there are few reports of leukemic synovitis and its prognosis is expected to be poor, we recommend performing arthrocentesis in leukemia patients with joint swelling in order to make a diagnosis of leukemic synovitis.

## Data Availability

The data used to support the findings of this study are included within the article.
